# Learning Health Systems Are Resilient Health Systems: Comment on "Re-evaluating Our Knowledge of Health System Resilience During COVID-19: Lessons From the First Two Years of the Pandemic"

**DOI:** 10.34172/ijhpm.8676

**Published:** 2024-09-07

**Authors:** Rachel Neill, Michael A. Peters

**Affiliations:** Health Systems Program, International Health Department, Johns Hopkins University Bloomberg School of Public Health, Baltimore, MD, USA.

**Keywords:** Learning Health Systems, Resilience, COVID-19, Health Policy, Pandemic Preparedness

## Abstract

Saulnier’s review, "Re-evaluating Our Knowledge of Health System Resilience During COVID-19: Lessons From the First Two Years of the Pandemic," analyzes health systems resilience in the first two years of the COVID-19 pandemic. A key finding was the importance of learning. In this commentary, we argue that strengthening systems-level learning capabilities could build resilient health systems. Drawing on learning theories and evidence from organizational resilience and management scholarship, we link the concept of learning loops with Blanchet’s resilience capacities framework, demonstrating the importance of higher levels of learning to build adaptive and transformative resilience capacities. We also argue for an increased focus on power analysis to analyze what is learned, who learns it, and who responds as determining factors to adaptation and transformation. Future research should empirically investigate the extent to which different types of learning supports – or impedes – the building of resilient health systems.

## Introduction

 Health systems resilience is the ability of a health system to absorb, adapt, and/or transform when experiencing an acute or chronic shock while retaining the basic function of service delivery.^1^ The COVID-19 pandemic offered the opportunity to study responses to a single shock at unprecedented scale, highlighting the promise and pitfalls of health systems resilience as a lens to prepare for, respond to, and recover from shocks.

 In 2023, Saulnier et al conducted a narrative review on the state of knowledge on health systems resilience in the first two years of the COVID-19 pandemic.^2^ Their review adopted Blanchet’s “Dimensions of Resilience Governance Framework” to analyze 63 articles on health systems resilience and the COVID-19 pandemic. They identified health systems resilience capacities across the framework’s categories of knowledge, uncertainty, legitimacy, and interdependence and describe how they influence health systems’ ability to absorb, adapt, and transform.^1,2^ Findings emphasized the critical importance of within-country and cross-country learning at all levels of the health system.^2^

 Their review also identified that most evidence on health systems resilience is on absorptive capacity (a health system’s ability to absorb a shock while maintaining its functioning with the same level of resources) and adaptative capacity (a health system’s ability to maintain functioning with fewer or different resources), with fewer insights on transformative capacity (changing the structure and functioning of the system in response to shock).^2^ Their findings and others point to an emerging consensus that “learning” is a core capability of health systems resilience.^2-4^ But critical questions remain, such as: what types of learning occurred during the COVID-19 pandemic, by who, and with what consequences? What is the relationship between different types of learning and different resilience capacities? And, why, despite the importance of learning, did learning rarely translate to transformation?

 The learning health systems literature can deepen our conceptual understanding of the relationship between learning and health systems resilience. In this commentary, we build on Saulnier’s findings to draw out connections between different types of learning and absorptive, adaptive, and transformative capacities. In doing so, we highlight what types of learning for health systems resilience are well documented, where the gaps remain, and offer critical questions for future research, policy, and practice with a particular focus on the relationship between learning, power, and transformation.

## What Is a Learning Health System?

 Learning “makes the link between past actions, the effectiveness of those actions, and future actions.”^5^ According to a commonly used definition from the Institute of Medicine, “In a learning healthcare system, science, informatics, incentives and culture are aligned for continuous improvement and innovation, with best practices seamlessly embedded in the delivery process, patients and families active participants in all elements, and new knowledge captured as an integral by‐product of the delivery experience.”^5^

 Learning is theorized as having different “loops” of feedback, which enable continuous evaluation, assessment, and improvement. Most fundamentally is single loop learning which supports change within an existing set of alternatives.^5,6^ This manifests in health systems as quality improvement initiatives, monitoring and evaluation, or operational research that can lead to programmatic changes without affecting the context in which health services are provided. Double loop learning begins to question assumptions and root causes of challenges, spurring more meaningful policy change.^5,6^ When policies are changed to solve systems bottlenecks, or new approaches are systematically adopted or scaled across a system, double loop learning may be occurring. Finally, triple loop learning challenges deeper assumptions and improves the ability of the system to learn in the future.^5^ Triple loop learning often entails deeper corrective changes to the conceptualization of the system itself and is considered the most difficult to attain and proactively plan for.^7^

 Single, double, and triple loop learning occur at all levels of the health system and happen through multiple pathways. These include from learning from information (eg, cognitive learning through routine information systems and research), learning via deliberation (eg, social learning including consensus building, stakeholder dialogue, and community engagement), and learning via action (“learning by doing”).^5^ A review of learning health systems in high-income countries identified four enabling conditions of learning health systems: data systems and informatics; a supportive organizational culture; a workforce skilled in principles of learning health systems; and resources dedicated for systems of learning.^8^ These enabling features are also likely relevant for optimizing pathways in low- and middle-income countries.

## The Relationship Between Learning Loops and Resilience Capacities

 One of the challenging aspects of health systems resilience as a concept is a lack of clarity on whether resilience is a process or an outcome. Applying the concept of learning loops can further elucidate how resilience can be both a process and an outcome simultaneously in a way that appreciates the emergent nature of complex adaptive systems and draws from evidence on how organizations learn.

 Applying this literature to Saulnier’s findings, we argue that single loop learning is most likely to support absorptive capacities. These examples emerge prominently from Saulnier’s review and tend to focus on individual-level learning in response to a shock, including action learning from healthcare workers to make decisions and improvise due to a lack of resources, and forms of learning through deliberation to engage stakeholders, distribute resources and coordinate responses.^2^

 Strengthening adaptive capacities is likely to be driven by double loop learning. Here, we identify more limited examples from Saulnier’s review; for example, how forms of information and action learning at the systems-level led to strengthening public health capabilities before and during the COVID-19 pandemic and how values and political economics changed to enable policy responses in a timely manner.^2^

 Other literature on health systems resilience and learning similarly describes double loop learning via adaptation. For example, Bishai and colleagues’ review on practical strategies to achieve resilience, suggests the potential of learning from information to build resilience, including the importance of evaluations and investing in “lateral learning” to benchmark performance and learn from within-country exemplars.^9^ A review of Guinea’s ability to learn from concurrent outbreaks also identified examples of double loop learning, especially learning through deliberation and information (via the creation of an Ebola response unit which later became a sustainable autonomous agency charged with epidemiological surveillance and health security and developing a “general states of health” forum).^10^ Finally, evidence points to missed opportunities for double loop learning and systems-level adaptation due to a lack of learning through limited dialogue with vulnerable populations (who often lack sufficient power to be at the decision making table).^11,12^

 Finally, we expect that triple loop learning is the most likely lead to transformative capacities, or transformation of the health system in response to a shock. Transformation is likely to require strong learning systems that are capable of challenging existing assumptions, contexts, and systems-level structures. Heath systems platforms for triple loop learning must therefore already be in place to understand when shocks or other contextual factors—whether climate change or artificial intelligence—necessitate a paradigm shift in the way learning is incorporated into decision-making. But the ability to challenge assumptions to spur triple loop learning is also impacted by health systems power dynamics, as those benefiting from the status quo may have little incentive to transform the system.^13^

 Despite the unprecedented scale of the COVID-19 pandemic, Saulnier et al identifies few examples of transformative capacities. This points to a possible gap in health systems’ ability to systematically learn in a dynamic, adaptive manner required to challenge assumptions and drive transformative change (when appropriate). Alternatively, it is possible that the underlying causes of inequities driving a lack of resilience – for example, a lack of broadband access for rural populations preventing access to new telemedicine adaptations, lack of health insurance for low-income populations, or increased risk of transmission in crowded migrant housing^2^ – were not addressed (at least in the first two years of the COVID-19 pandemic), limiting transformation Saulnier’s review therefore highlights much more evidence on informational learning and the documentation of inequities than the transformative actions needed to address them.

## Can Single, Double, and Triple Loop Learning Be a Pathway to Building Health Systems Resilience? Outstanding Questions and Implications for Future Research

 There is a growing consensus on the importance of learning as a pathway to building resilience, particularly the role of double loop learning in strengthening adaptive capacities. Moving forward, health systems researchers can expand on Saulnier’s findings by using organizational learning and resiliency theories to deepen the understanding of how, why, when, and for whom does learning lead to the building of absorptive, adaptive, and transformative resilience capabilities. And under what conditions are different types of learning facilitated? Key to this is centering the role of power dynamics in determining the type of learning that occurs, what types of information is considered legitimate, and if learning translates to systems-level change.

 One area ripe for empirical inquiry is whether different types of shocks necessitate different types of learning. When is a shock severe enough to necessitate adaption compared to absorption – and does this change based on who is impacted most by the shock? When is triple loop learning and transformation likely to be required compared to more incremental adaptation or a conscious decision to avoid transformation and keep a stable system? Similar to critiques on the normative preference for triple loop learning as the “highest attainment” in the organizational learning literature,^7^ careful attention should be paid to uncritical evangelism for transformation as a normative good.Experience of countries learning from past shocks to improve future responses, for example, may entail gradual adaptation based on action learning which, over time, strengthens the health system’s preparedness and response functions in an incremental, yet meaningful, manner.^10^ These examples of double loop learning are likely to underpin the building of everyday resilience in a manner that can strengthen the health system over time.

 Another question is the empirical relationship between learning health systems and the capacities of resilient health systems. As learning capabilities are strengthened, will resilience capacities increase as a result? Orth and Schuldis empirically tested the relationship between organizational learning and resilience in Central Europe, demonstrating a strong relationship between organizational learning capabilities and adaptive capacities.^14^ Evidence from Sauliner’s review points to the importance of both informational learning (eg, from information systems and risk analysis) and learning through deliberation (eg, via community engagement, consultative governance, and bottom-up communication).^2^ Future research can expand empirical analysis on the conditions that facilitate learning loops, the types of learning required and which actor(s) need to be involved, providing stronger evidence for investments in learning as a vehicle to strengthen resiliency.

 Finally, both learning health systems and health systems resilience have been subjected to criticism for their lack of explicit recognition of power and politics.^3,13^ Translating learning into transformative change will require a critical interrogation of the deeper values and socio-economic structures of health systems. As Saulnier indicated, equity is increasingly considered as a component of health systems resilience^2^; how can different types of learning support the achievement of equitable health systems, rather than a reinforcement of the status quo? The incorporation of power analysis into health systems resilience scholarship is critical to interrogating these dynamics.

## Conclusion

 Saulnier highlights that, “Although the necessity to integrate and use multiple forms and sources of information is clear [during a shock], it is still unclear which sources and forms are most influential on the system’s ability to respond and its capacity for resilience.”^2^ We argue that the missing piece of this puzzle is the ability of the health system to engage in higher-level learning to spur adaptation and/or transformative change. More explicit incorporation of different types of learning as critical drivers towards building absorptive, adaptive, and transformative capacities can help develop operational guidance on how to build resilience in health systems. Health systems must advance beyond single loop learning to actively enable double and triple loop learning to adapt and transform in the face of both acute shocks and chronic stressors. Increased attention to equity and power dynamics in health systems is required to ensure that adaptation and transformation improves the health systems’ ability to generate equitable outcomes for all populations.

## Acknowledgements

 The authors wish to thank the anonymous reviewer whose comments substantively improved the commentary.

## Ethical issues

 Not applicable.

## Conflict of interests

 Authors declare that they have no conflict of interests.
